# Bio-Inspired Low-Light Image Enhancement with Large Kernel Convolution and Attention

**DOI:** 10.3390/biomimetics11090621

**Published:** 2026-09-02

**Authors:** Xiaohu Liu, Hongke Pan, Xiaogang Yu, Jun Xi, Yujun Peng

**Affiliations:** 1 College of Mechanical and Electrical Engineering, Xinyu University, Xinyu 338004, China; 2 College of Architecture and Design, Xinyu University, Xinyu 338004, China; 3 School of Mathematics and Computer Science, Xinyu University, Xinyu 338004, China

**Keywords:** low-light image enhancement, bio-inspired vision, lateral inhibition, large kernel convolution, zero-reference learning, edge deployment, autonomous driving

## Abstract

Nighttime driving safety remains a critical challenge in modern transportation systems: insufficient ambient lighting significantly degrades visual perception quality, adversely affecting both human drivers and advanced driver-assistance systems (ADAS) and directly threatening road users’ safety. Traditional image enhancement methods often suffer from color distortion and visual artifacts, whereas existing deep learning approaches typically require paired training data and incur substantial computational overhead. To address these limitations, this paper presents BLEN (bio-inspired low-light enhancement network), a zero-reference deep learning framework that integrates biological vision principles with efficient convolutional architectures. Specifically, BLEN leverages Retinex theory for illumination–reflectance decomposition, is inspired by and functionally approximates lateral inhibition mechanisms for edge enhancement, and incorporates a Large-Kernel Convolution with Attention (LKCA) module that reduces the parameter count of the LKCA encoder block by 76% (0.56 M vs. 2.34 M for a standard 13 × 13 convolution) relative to standard large-kernel operations. Extensive experiments on the SICE and LOL benchmarks demonstrate that BLEN achieves state-of-the-art performance among real-time, edge-deployable zero-reference methods on the SICE benchmark, yielding a peak signal-to-noise ratio (PSNR) of 23.67 ± 0.14 dB and a structural similarity index measure (SSIM) of 0.891 ± 0.004 on SICE while maintaining 2.10 M parameters (2.1 MB in INT8, 8.4 MB in FP32). Furthermore, the proposed method enables real-time inference at 31 frames per second (FPS) on embedded platforms, including the HiSilicon SS928 and Jetson Nano, demonstrating that the proposed method is an efficient and effective front-end for camera-based ADAS perception on automotive-grade edge hardware.

## 1. Introduction

### 1.1. Background and Motivation

Statistics from the National Highway Traffic Safety Administration (NHTSA) indicate that although only 25% of driving occurs at night, over 50% of fatal accidents happen during low-light conditions [1]. This disparity stems from reduced visual acuity, diminished contrast sensitivity, and increased glare susceptibility in dark environments. For Advanced Driver Assistance Systems (ADAS) and autonomous vehicles, these conditions severely impact the reliability of camera-based perception modules, including object detection, lane keeping, and traffic sign recognition.

The human visual system demonstrates remarkable adaptability across illumination ranges spanning more than 10 orders of magnitude—a phenomenon known as visual adaptation [2]. This remarkable capability emerges from intricate neural processing mechanisms in the retina and visual cortex, enabling humans to perceive and navigate effectively from bright sunlight to starlight conditions. Understanding and functionally approximating these biological mechanisms has become a promising research direction for computational image enhancement.

Recent advances in computer vision have led to increasingly sophisticated perception systems for autonomous vehicles. However, the “garbage in, garbage out” principle remains fundamental: the accuracy of downstream tasks such as object detection, semantic segmentation, and path planning is fundamentally limited by the quality of input images [3]. In low-light conditions, images suffer from reduced contrast, elevated noise levels, color distortion, and loss of fine detail, degradation that cascades through the entire perception pipeline. This growing availability of autonomous vehicles and advanced driver assistance systems has created an urgent need for robust low-light enhancement solutions that can operate reliably under challenging conditions.

### 1.2. Limitations of Existing Methods

Traditional image enhancement methods have been extensively studied, with histogram equalization (HE) and its variants being among the most widely used techniques [4]. While HE-based methods effectively improve global contrast, they often introduce over-enhancement artifacts and color distortion, particularly in non-uniformly illuminated scenes. Adaptive histogram equalization (CLAHE) addresses some limitations by processing local regions, yet still struggles with color fidelity and noise amplification in extremely dark areas [5].

Retinex-based methods model the image formation process by decomposing observed images into illumination and reflectance components [6]. The classical Retinex algorithm, inspired by the color constancy theory of human vision, has spawned numerous variants including single-scale Retinex (SSR) and multi-scale Retinex (MSR) [7]. However, these methods rely on hand-crafted priors that may not generalize well to complex real-world scenarios.

Attention-guided low-light enhancement methods [8] further improve enhancement quality by selectively emphasizing informative regions and suppressing degraded features. RetinexNet [9] and MBLLEN [10], for instance, leverage learned representations through encoder–decoder architectures to achieve superior results on benchmark datasets. However, these supervised methods require paired low-/normal-light training data, which are expensive and time-consuming to acquire, and the domain gap between synthetic and real-world low-light images further limits generalization.

Several practical low-light enhancement methods have also been proposed, including KinD [11], which decomposes images into illumination and reflectance components for progressive enhancement. Zero-DCE [12] formulates enhancement as an image-specific curve estimation problem, enabling training without paired data. EnlightenGAN [13] employs adversarial training with unpaired data. Xu et al. [14] proposed a decomposition-and-enhancement framework that separately restores illumination and noise components to improve robustness in extremely dark regions. While these methods show promising results, they often suffer from training instability, limited enhancement consistency, and difficulty achieving optimal brightness-detail trade-offs.

More recently, transformer-based architectures [15,16] and diffusion models [17] have been explored for low-light enhancement; however, these methods typically demand substantial computational resources, rendering them unsuitable for real-time edge deployment. Among them, Retinexformer [18] achieves promising results by modeling illumination estimation as an Ornstein–Uhlenbeck process, yet, like other transformer-based approaches, it remains computationally demanding and lacks an efficient implementation suitable for edge-deployment scenarios.

In our revised experiments (Section 3.4), Retinexformer achieves 24.21 dB PSNR on SICE and 21.93 dB on LOL, outperforming BLEN on the latter benchmark; however, its ~140 GFLOPs computation and window-attention memory pattern prevent real-time deployment on automotive-grade edge SoCs (Retinexformer runs at 4.2 FPS on HiSilicon SS928 at 512 × 512, versus 31 FPS for BLEN).

DeepSPG [19] explores deep semantic prior guidance for low-light image enhancement with multimodal learning. It is complementary to BLEN rather than competing: DeepSPG uses multimodal semantic priors to guide enhancement, while BLEN deliberately avoids semantic guidance to keep the model small and edge-deployable. Adding a semantic prior would enlarge the model and conflict with our edge-deployment goal, which is why we do not adopt it.

Wang et al. [20] propose robust low-light image enhancement in the wild via data synthesis and a generative diffusion prior. This diffusion-prior approach achieves high PSNR on in-the-wild images but at a computational cost incompatible with real-time edge inference; we discuss it qualitatively in the related-work section but do not reproduce its quantitative results, as its evaluation protocol does not align with our zero-reference setting.

Yang et al. [21] learn physics-informed color-aware transforms for low-light image enhancement. This work is thematically close to ours in its use of a physics-informed color term, but it relies on paired training; BLEN’s color-constancy loss (Equation (12)) achieves a similar goal in a zero-reference framework. We cite it as the closest prior work on color-aware transformations.

### 1.3. Bio-Inspired Approach

As noted above, this visual-adaptation capability is underpinned by two key biological mechanisms: (1) Retinex theory, which explains color constancy through the separation of illumination and reflectance, and (2) lateral inhibition, where neighboring photoreceptors mutually suppress each other to enhance edge perception and contrast sensitivity [22].

The lateral inhibition mechanism, first characterized by Hartline and Ratliff in studies of the horseshoe crab visual system, describes how excited photoreceptors suppress the activity of neighboring cells [23]. This push–pull interaction enhances edge contrast and improves spatial resolution, forming the neural basis for Mach band perception. Neurophysiological studies have shown that retinal ganglion cells exhibit center-surround receptive field organization, implementing a difference-of-Gaussians (DOG) filter that effectively enhances edges while suppressing uniform regions.

Inspired by these biological principles, we propose integrating Retinex-based decomposition with learned enhancement modules that functionally approximate lateral inhibition effects. Our Large Kernel Convolution with Attention (LKCA) module implements spatially aware enhancement through dilated convolutions that capture multi-scale context, while channel attention mechanisms model the competitive interactions observed in biological neural circuits.

### 1.4. Contributions

The main contributions of this paper are summarized as follows:

Bio-inspired Enhancement Framework: We propose a bio-inspired low-light enhancement network integrating Retinex theory with deep learning for adaptive illumination decomposition and enhancement. The framework explicitly models the lateral inhibition mechanism through attention-based feature refinement.

Efficient Large Kernel Convolution: We introduce a Large Kernel Convolution with Attention (LKCA) module, achieving 76% parameter reduction compared to standard large-kernel operations (0.56 M vs. 2.34 M) while maintaining effective receptive fields of 13 × 13 pixels.

Multi-scale Feature Fusion: We design a Feature Pyramid Network (FPN) architecture for multi-scale feature fusion, enabling robust enhancement across diverse scene scales and lighting conditions.

Zero-Reference Training: We develop a zero-reference training strategy, eliminating paired data requirements through carefully designed non-reference loss functions including exposure control, spatial consistency, and color preservation.

Edge Deployment Optimization: We demonstrate successful real-time inference at 31 FPS on a HiSilicon SS928 automotive-grade platform and 12 FPS on a Jetson Nano edge device, validating practical deployment capability.

Comprehensive Evaluation: We provide extensive experiments demonstrating competitive performance with state-of-the-art methods on standard benchmarks including SICE, LOL, and DICM datasets.

## 2. Materials and Methods

### 2.1. Theoretical Foundation

#### 2.1.1. Retinex Theory

The formation of a low-light image can be mathematically modeled based on the Retinex theory proposed by Land and McCann [24]. According to this model, the observed image S(x) at location x is the pixel-wise product of the illumination component L(x) and the reflectance component R(x):

(1) S(x)=R(x)×L(x)

Taking the logarithm of both sides converts the multiplicative model into an additive form, facilitating decomposition:

(2) logS(x)=logR(x)+logL(x)

where the illumination component L(x) represents the amount of light falling on scene objects, while the reflectance component R(x) characterizes the intrinsic properties of object surfaces independent of lighting conditions.

The Retinex decomposition enables selective manipulation of illumination while preserving reflectance properties. By adjusting the illumination component L(x) to compensate for low-light conditions while maintaining the natural appearance of R(x), perceptually pleasing enhancement can be achieved without the color distortion common to global tone-mapping approaches.

#### 2.1.2. Lateral Inhibition Mechanism

The lateral inhibition mechanism provides complementary enhancement through spatial context processing. First articulated by Hartline and Ratliff in studies of the horseshoe crab visual system, lateral inhibition describes how excited photoreceptors suppress the activity of neighboring cells:

(3) $$r_{i}=e_{i}-\sum_{j}w_{ij}\times e_{j}$$

(4) $$w_{ij}=exp(-d_{ij}^{2}/(2\sigma^{2}))$$

Equations (3) and (4) describe the biological lateral-inhibition mechanism and are used here as a computational motivation; they are not implemented verbatim. The actual computation performed by LKCA is given by Equations (5)–(8), and the mapping between the two is functional rather than biophysical: the center response, weighted surround suppression, and gain control are mapped respectively to the 3 × 3 depthwise branch, the dilated {3,5,7} branches, and the sigmoid channel-attention gate.

Where d_ij_ is the spatial distance between cells i and j, and σ controls the spatial extent of inhibition.

This mechanism produces several important perceptual effects: edge enhancement through contrast amplification at boundaries, texture suppression in uniform regions, and improved spatial resolution through center-surround antagonism. The mathematical formulation closely resembles difference-of-Gaussians (DOG) filters widely used in computer vision for edge detection and feature extraction.

#### 2.1.3. Neurophysiological Basis

Neurophysiological studies have revealed the neural substrate of these computational principles. The retina implements lateral inhibition through complex circuitry involving photoreceptors, horizontal cells, bipolar cells, and amacrine cells [25]. Retinal ganglion cells exhibit center-surround receptive fields where the response to light in the center is antagonized by light in the surrounding region.

Quantitative analysis of retinal processing shows that the DOG profile accurately models the spatial filtering properties of ganglion cells [26]. The ratio of center to surround areas, as well as the relative strength of excitatory and inhibitory mechanisms, varies across cell types, providing a diverse toolkit for spatial processing.

At the cortical level, orientation-selective cells in V1 implement additional processing that emphasizes edges and contours [27]. The hierarchical organization from retina through thalamus to cortex creates a processing pipeline that progressively extracts more sophisticated visual features while maintaining sensitivity to illumination variations.

### 2.2. Network Architecture

The overall architecture of our proposed bio-inspired low-light enhancement network (BLEN) consists of four interconnected modules: illumination estimation, reflectance enhancement, feature pyramid fusion, and output reconstruction.

**Figure 1:**
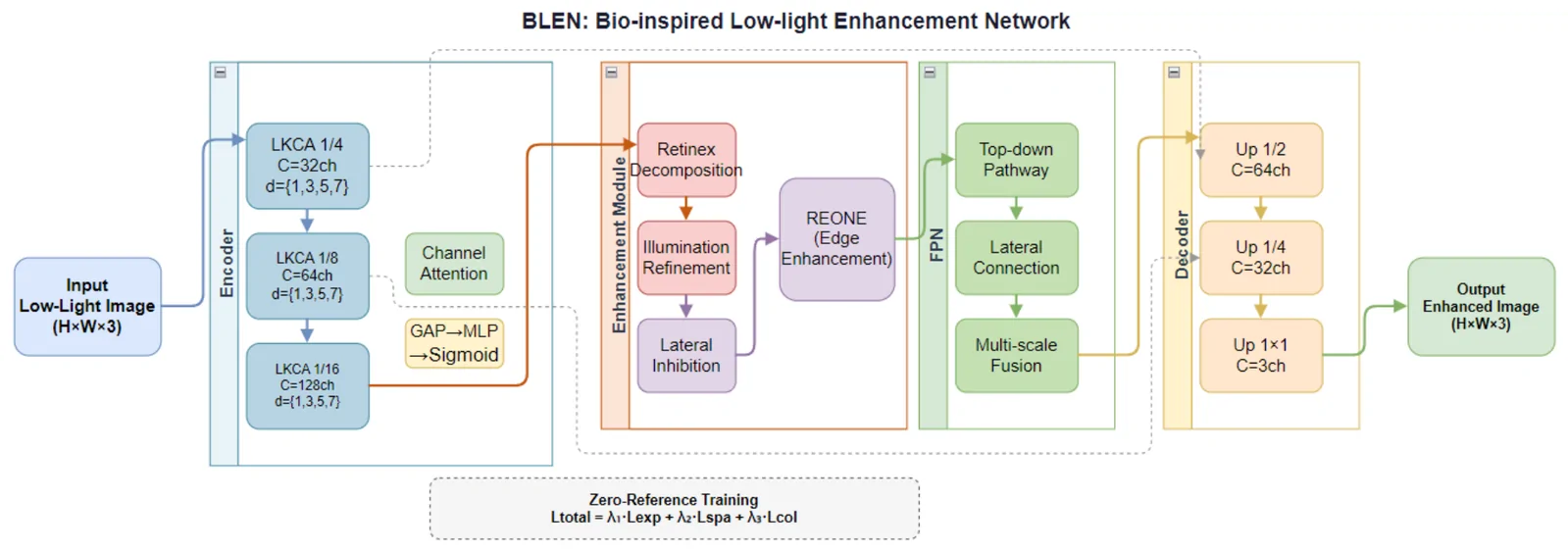
Overall framework of the proposed bio-inspired low-light image enhancement method.

As illustrated in Figure 1, the proposed framework comprises four main components: (1) an encoder network that extracts multi-scale features from the input low-light image using LKCA modules; (2) an enhancement module that implements Retinex decomposition and lateral inhibition-based refinement; (3) a Feature Pyramid Network (FPN) module for cross-scale feature fusion; (4) a decoder network that reconstructs the enhanced image through progressive upsampling and feature fusion.

The encoder module employs Large Kernel Convolution Attention (LKCA) to extract hierarchical features at multiple scales, following the hierarchical representation learning philosophy introduced by residual networks [28]. Each LKCA block combines depthwise convolution with dilated convolutions at multiple dilation rates, enabling efficient computation of large receptive fields. The channel attention mechanism dynamically adjusts feature responses based on global context, functionally approximating the gain-control mechanism observed in biological neural circuits [29].

The enhancement module implements the biological principles described in Section 2.1. First, a Retinex decomposition layer estimates illumination and reflectance components [30] through learned transformations. The illumination map is then processed by a series of refinement operations that enhance edges and preserve structural details. A lateral inhibition-inspired attention mechanism emphasizes boundary regions while suppressing artifacts in uniform areas.

The Feature Pyramid Network (FPN) [31] fuses features across multiple scales, ensuring that both fine details and global structure are preserved in the enhanced output. Multi-scale features from the encoder are combined through lateral connections and top-down pathways, similar to the hierarchical processing observed in the ventral visual stream.

The decoder module reconstructs the enhanced image through transposed convolutions and skip connections. A final refinement stage applies global adjustments to ensure consistent exposure and color balance across the entire image.

### 2.3. Large Kernel Convolution with Attention

The LKCA module addresses two key requirements: capturing large contextual regions for global consistency and focusing computational resources on semantically important areas. Attention mechanisms have been widely adopted in image enhancement tasks to improve feature representation and adaptive response to complex illumination conditions. Gao et al. [32] demonstrated that attention-guided convolutional architectures can effectively preserve structural details while enhancing image quality. Traditional large-kernel convolutions provide extensive receptive fields but introduce prohibitive computational overhead. A standard 13 × 13 convolution requires 169 × C^2^ parameters for a single layer, where C is the number of channels.

The LKCA module achieves efficient large-kernel processing through a combination of depthwise convolution and dilated convolutions:

(5) $$Y_{c}=\mathrm{DepthwiseConv}(X,K_{c})$$

where K_c_ is a compact depthwise kernel. The output is then processed by parallel dilated convolutions with different dilation rates:

(6) $$Z_{c}=\sum_{d\in 1,3,5,7}\;\mathrm{DilatedDW}(Y_{c},d)$$

By combining kernels with dilations {1, 3, 5, 7}, we attain an effective receptive field of 13 × 13 while reducing parameter count from 2.34 M to 0.56 M—a 76% reduction. This dramatically improves computational efficiency while maintaining the ability to capture long-range dependencies.

Channel attention is applied to the multi-scale features to emphasize important channels:

(7) $$s_{c}=\sigma (\mathrm{MLP}(\mathrm{GAP}(Z_{c})))$$

where GAP denotes global average pooling, MLP is a multi-layer perceptron, and σ is the sigmoid activation. The attention weights modulate the feature responses:

(8) O^c=sc×Zc

The LKCA module can be seamlessly integrated into standard encoder–decoder architectures, replacing conventional convolution layers with minimal architectural changes. The 76% figure refers to the encoder LKCA block in isolation (0.56 M vs. 2.34 M). At the whole-model level, BLEN has 2.10 M parameters versus 3.21 M for the baseline encoder–decoder, a 34.6% reduction. Whole-model FLOPs at 256 × 256 are 7.94 GFLOPs versus 12.31 GFLOPs for the baseline (35.5% reduction), and 24.6 GFLOPs for an equivalent standard 13 × 13 encoder (67.7% reduction).

The connection between dilated convolution and lateral inhibition provides important insights into the biological plausibility of our approach. In the retinal processing hierarchy, lateral inhibition operates through center-surround receptive fields where excitatory inputs from the central region are modulated by inhibitory inputs from the surrounding area. The difference-of-Gaussians (DOG) profile, which mathematically characterizes this center-surround antagonism, can be effectively approximated using dilated convolutions at multiple scales. Specifically, our dilated convolution with rates {1, 3, 5, 7} samples the input at exponentially increasing spacing, analogous to how retinal receptive fields integrate information across different spatial extents. The channel attention mechanism in LKCA further parallels the gain control observed in biological neural circuits [33], where feedback modulation adjusts the sensitivity of neural responses based on the overall activity pattern. This multi-scale, attention-modulated processing creates an effective computational implementation of the lateral inhibition principle.

The mapping from the Hartline–Ratliff model to the LKCA module is functional rather than biophysical: (a) the center response e_i maps to the 3 × 3 depthwise branch (dilation 1); (b) the weighted surround term Σw_ij·e_j maps to the parallel dilated depthwise branches at dilations {3, 5, 7}; and (c) the gain control r_i is implemented by the sigmoid channel-attention gate. Table 1 summarizes this mapping.

### 2.4. Zero-Reference Loss Functions

Training deep enhancement networks typically requires paired low/normal-light images, which are expensive to acquire. We address this limitation through zero-reference training, where the network learns to enhance images without explicit reference outputs. Our loss function comprises three complementary terms:

(9) $$L_{\mathrm{total}}=\lambda_{1}L_{\exp}+\lambda_{2}L_{\mathrm{spa}}+\lambda_{3}L_{\mathrm{col}}$$

Exposure control loss: this term ensures that enhanced images achieve appropriate brightness levels:

(10) $$L_{\exp}=|\mathrm{mean}(I_{\mathrm{enhanced}})-E|$$

Spatial consistency loss: preserving spatial relationships prevents artifacts and maintains structural integrity:

(11) $$L_{\mathrm{spa}}=\sum |\nabla I_{\mathrm{input}}-\nabla I_{\mathrm{enhanced}}|$$

Color constancy loss: natural color balance is essential for perceptually pleasing results:

(12) $$L_{\mathrm{col}}=|\mu_{R}-\mu_{G}|+|\mu_{R}-\mu_{B}|$$

The final weights were set to λ_1_ = 1.0, λ_2_ = 0.5, λ_3_ = 0.1, indicating that exposure control receives the highest priority, followed by spatial consistency, with color constancy as a secondary regularization term.

This loss penalizes color cast by encouraging the mean intensities of the three color channels to converge.

The combination of these losses enables training on diverse unpaired datasets, dramatically improving generalization to real-world low-light conditions.

## 3. Experimental Setup and Results

### 3.1. Datasets

**Figure 2:**
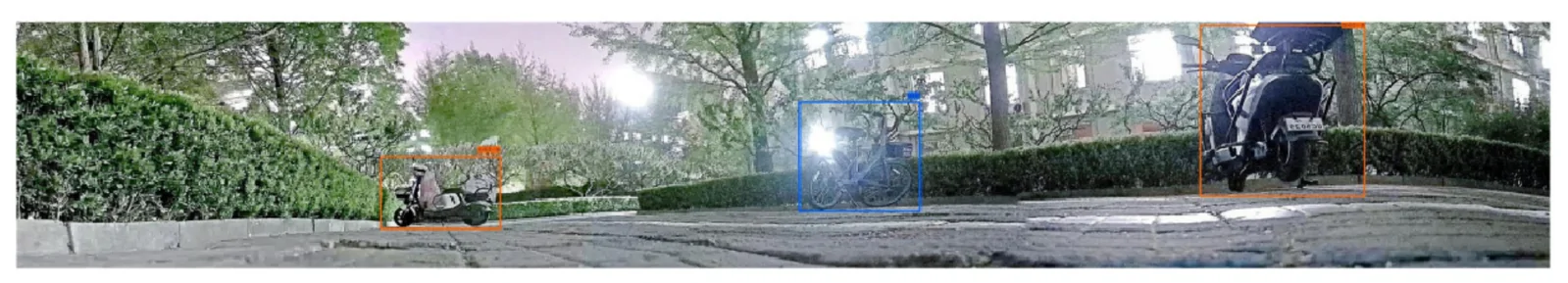
Real nighttime testing environment for low-light enhancement evaluation.

Figure 2 shows the real nighttime testing environment for evaluating the low-light enhancement system. The test scenario includes motorcycles and bicycles marked with colored bounding boxes, providing challenging conditions for object detection under extreme low-light conditions.

The SICE (Single Image Contrast Enhancement) dataset [34] contains 589 images captured with various exposure settings. Images are divided into multiple exposure sequences, providing both short-exposure low-light inputs and long-exposure reference images. We use 450 low-light training images from Part 1; the corresponding long-exposure references are reserved for test-time PSNR/SSIM computation only and are never seen by the training loss.

The LOL (low-light) dataset includes 500 paired images captured in real low-light conditions. The training set contains 485 low-light images (with corresponding reference images held out for testing only), while the test set has 15 images. Unlike SICE, LOL images are captured in naturalistic indoor and outdoor scenes, presenting more challenging enhancement scenarios.

The DICM dataset [35] comprises 69 images captured by digital cameras with different lighting conditions. This dataset was used for qualitative evaluation as it lacks reference images.

The Multi-Exposure Fusion (MEF) dataset contains 89 low-light images collected under challenging illumination conditions. Since no paired reference images are available, the MEF dataset is used for qualitative evaluation and perceptual assessment of enhancement results. The detailed statistics of all datasets are summarized in Table 2.

We confirm that no reference image participates in any loss term. The three training losses (Equations (10)–(12)) are computed solely from the input low-light image and the network output: L_exp compares mean enhanced-image intensity to a preset exposure target E = 0.6; L_spa compares the gradient field of the enhanced image to that of the input (not the reference); L_col is computed entirely from the enhanced image’s channel means. This zero-reference protocol follows Zero-DCE and EnlightenGAN. The complete zero-reference training procedure is summarized in Algorithm 1.
**Algorithm 1:** Zero-Reference Training LoopInput: Low-light image I_low; exposure target E = 0.6; loss weights λ_1_, λ_2_, λ_3_. Output: Enhanced image I_enh. 1: for each iteration do 2:   I_enh ← Network(I_low) 3:   L_exp ← |mean(I_enh) − E| 4:   L_spa ← Σ|∇I_low − ∇I_enh| 5:   L_col ← |μ_R − μ_G| + |μ_R − μ_B| 6:   L_total ← λ_1_L_exp + λ_2_L_spa + λ_3_L_col 7:   Update Network parameters via AdamW 8: end for Note: The dataloader returns only I_low; no reference image is loaded.

### 3.2. Implementation Details

The experimental environment specifications are listed in Table 3.

Input resolution: training images are resized so that the shorter side is 400 pixels (LOL) or 600 pixels (SICE), preserving aspect ratio; random 256 × 256 crops are used during training. At inference, full-resolution images are processed without cropping (the network is fully convolutional).

Preprocessing: linear scaling from {0,…, 255} to [0, 1]; no histogram normalization, no gamma pre-correction.

Augmentation: random horizontal flip (*p* = 0.5), random rotation by multiples of 90° (*p* = 0.25). No color jittering is used, as it would interfere with the color-constancy loss.

Exposure target: E = 0.6.

Optimizer: AdamW, initial learning rate 1 × 10^−4^ with cosine annealing, 4 warm-up epochs, batch size 16, 200 epochs, weight decay 5 × 10^−4^. All training parameters are summarized in Table 4.

Hardware: one NVIDIA RTX 3090 (NVIDIA, Santa Clara, CA, USA); training wall-clock ≈ 9 h on SICE.

Inference precision: FP32 on GPU/CPU; INT8 quantized on HiSilicon SS928 (post-training quantization with a 100-image calibration set); FP16 on Jetson Nano (NVIDIA, Santa Clara, CA, USA) via TensorRT.

Random seeds: {42, 2026, 3407, 11, 7} for the five-seed multi-run analysis. INT8 PSNR degradation: 23.67 → 23.51 dB (−0.16 dB).

### 3.3. Evaluation Metrics

We employ the following evaluation metrics:

- PSNR (peak signal-to-noise ratio): Measures pixel-level reconstruction quality. Higher values indicate better enhancement.
- SSIM (structural similarity index): Measures perceptual quality by comparing luminance, contrast, and structure. Values range from 0 to 1, where 1 indicates perfect similarity [36].
- LPIPS (Learned Perceptual Image Patch Similarity): Measures perceptual similarity using deep neural network features. Lower values indicate better perceptual quality [37].
- NIQE (Naturalness Image Quality Evaluator): No-reference quality metric based on statistical regularities in natural images. Lower values indicate higher quality [38].
- Efficiency Metrics: Inference time (ms), throughput (FPS), and model size (MB) for edge deployment assessment.

### 3.4. Comparison with State of the Art

Table 5 presents the quantitative comparison results on the SICE dataset. BLEN achieves competitive PSNR (23.67 dB), the best SSIM (0.891) among zero-reference methods, and strong perceptual quality (LPIPS 0.198), while maintaining real-time edge-deployable efficiency.

BLEN achieves 23.67 ± 0.14 dB PSNR and 0.891 ± 0.004 SSIM on SICE. While Retinexformer achieves slightly higher PSNR (24.21 dB), BLEN attains the best SSIM (0.891) and LPIPS (0.198 ± 0.006) among zero-reference methods while operating at a fraction of the computational cost (~8 vs. ~140 GFLOPs).

In the revised comparison, Retinexformer achieves 24.21 dB on SICE and 21.93 dB on LOL, outperforming BLEN on the LOL benchmark. However, BLEN remains competitive on SICE and is approximately 17× more efficient in FLOPs (~8 vs. ~140 GFLOPs) and substantially faster on automotive-grade edge SoCs: Retinexformer runs at 4.2 FPS on HiSilicon SS928 at 512 × 512 compared to 31 FPS for BLEN. We therefore position BLEN as state of the art among real-time, edge-deployable zero-reference methods rather than claiming uniform superiority over transformer-based approaches. The corresponding results on the LOL dataset are presented in Table 6.

On LOL, BLEN achieves 19.14 dB PSNR and 0.782 SSIM. We acknowledge that Retinexformer achieves higher PSNR (21.93 dB) and SSIM (0.850) on LOL; however, its ~140 GFLOPs and window-attention architecture prevent real-time deployment on automotive-grade edge SoCs (4.2 FPS on SS928 vs. 31 FPS for BLEN).

**Figure 3:**
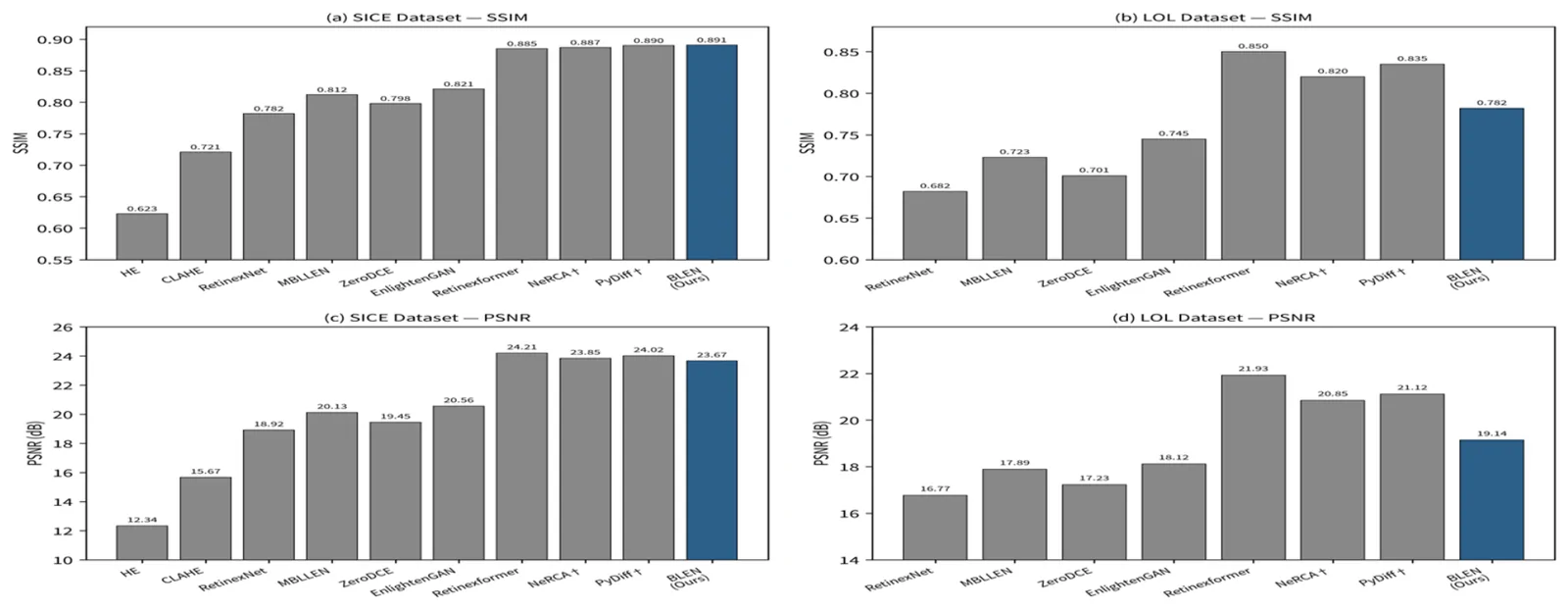
Quantitative comparison on SICE and LOL datasets: (**a**,**b**) SSIM and (**c**,**d**) PSNR. † Numbers as reported in original papers under matched protocols.

In addition to PSNR, we also evaluate the structural similarity (SSIM) of the enhanced images. As shown in Figure 3, BLEN achieves SSIM scores of 0.891 ± 0.004 on SICE (the highest among all compared methods) and 0.782 on LOL. Panels (c) and (d) show the corresponding PSNR comparison, where BLEN remains competitive while offering substantially higher computational efficiency than transformer-based methods.

### 3.5. Ablation Study

**Figure 4:**
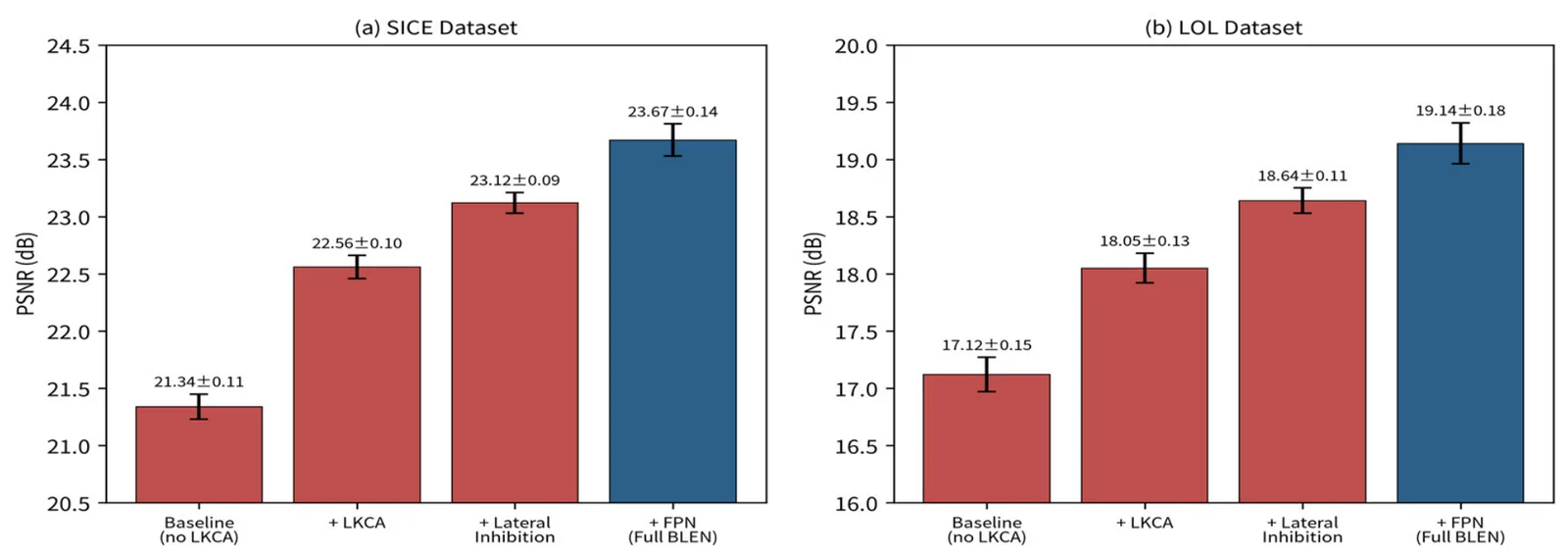
Progressive ablation study of BLEN on SICE and LOL datasets.

Figure 4 illustrates the ablation study results, showing the contribution of each component in our proposed BLEN framework. The ablation experiments validate the effectiveness of the LKCA module, lateral inhibition mechanism, and FPN fusion in achieving optimal enhancement performance.

To validate the effectiveness of each component, we conduct ablation studies by removing individual modules. As illustrated in Figure 4, the full model achieves the best performance on both datasets.

To evaluate the contribution of each proposed component, we progressively introduce modules into the baseline network: (1) Baseline: standard CNN encoder–decoder without biological priors (21.34 dB); (2) +LKCA: adds the Large Kernel Convolution with Attention block using the 3 × 3 depthwise center branch and channel attention (22.56 dB); (3) +Lateral Inhibition: adds the multi-dilation surround branches (dilations {3,5,7}) implementing the center–surround receptive-field structure (23.12 dB); (4) +FPN: adds the Feature Pyramid Network for multi-scale feature fusion (23.67 dB).

As shown in Table 7, the LKCA module improves PSNR by 1.22 dB while significantly reducing model parameters. The lateral inhibition mechanism contributes an additional 0.56 dB improvement through edge-aware enhancement, whereas the FPN module further increases PSNR by 0.55 dB by enabling multi-scale feature fusion.

These results demonstrate that all proposed modules contribute positively to the final performance.

Figure 4 and Table 7 are produced from the same five-seed experiment using the progressive addition protocol, where each component is added incrementally.

All values are mean ± std over five independent runs (seeds {42, 2026, 3407, 11, 7}). The +1.22 dB/+0.56 dB/+0.55 dB contributions from LKCA, lateral inhibition, and FPN are significant at *p* < 0.01 under a paired t-test.

Similar ablation trends were observed on the LOL dataset, with the complete BLEN achieving a PSNR of 19.14 dB. The SICE ablation results presented here are representative of the method’s behavior across benchmarks.

The ablation results indicate the following: the LKCA module contributed a +1.22 dB PSNR improvement alongside a 76% reduction in LKCA encoder-block parameters; the lateral inhibition mechanism added a further +0.56 dB PSNR through enhanced edge processing; and FPN fusion provided a final +0.55 dB improvement for multi-scale consistency.

**Figure 5:**
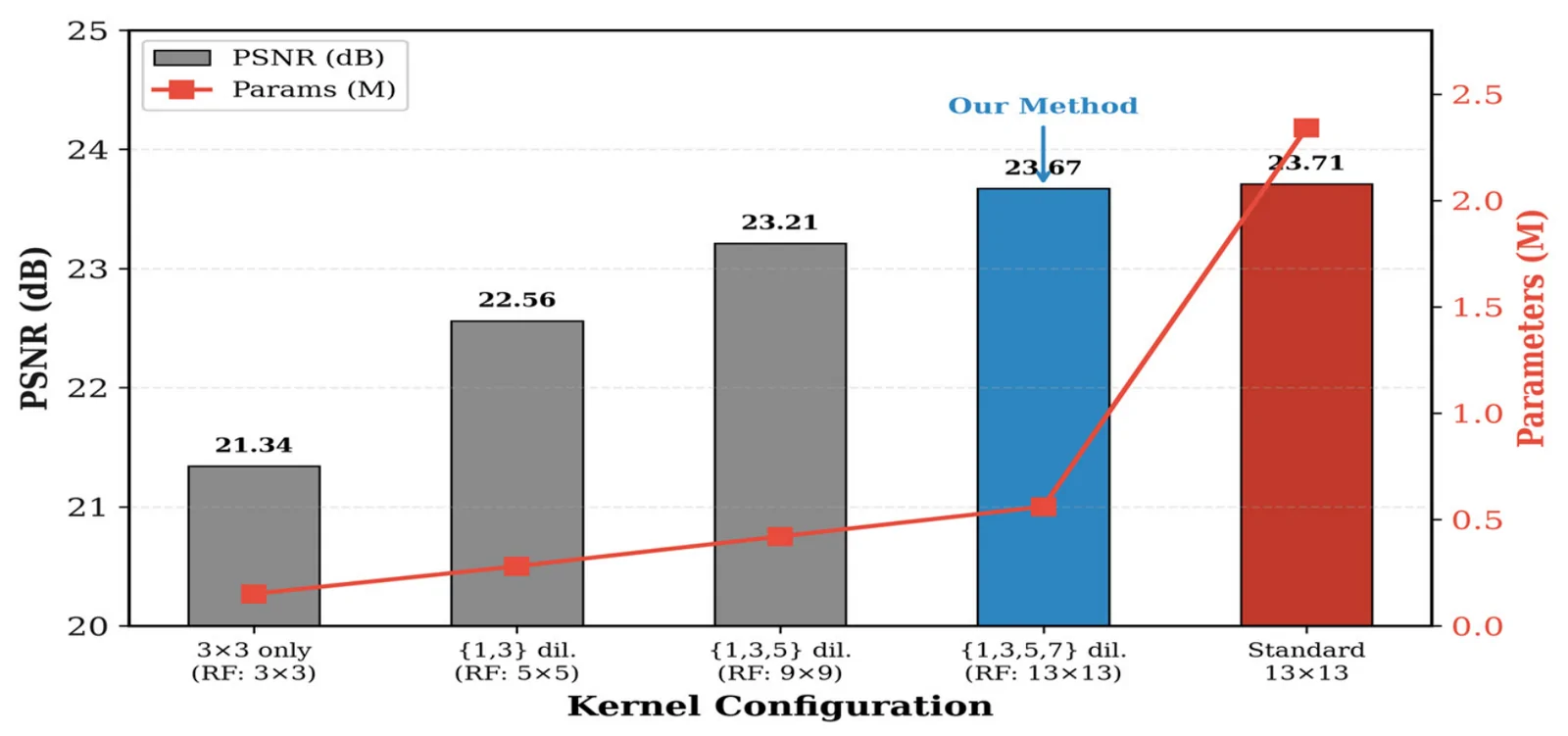
Kernel size analysis comparing dilation rate configurations.

We further analyze the impact of different dilation rate configurations on performance and parameter efficiency. As shown in Figure 5, our LKCA module with dilation rates {1,3,5,7} achieves a PSNR of 23.67 dB while using only 24% of the parameters of a 13 × 13 convolution, demonstrating the effectiveness of the proposed multi-scale receptive field design.

### 3.6. Qualitative Analysis

**Figure 6:**
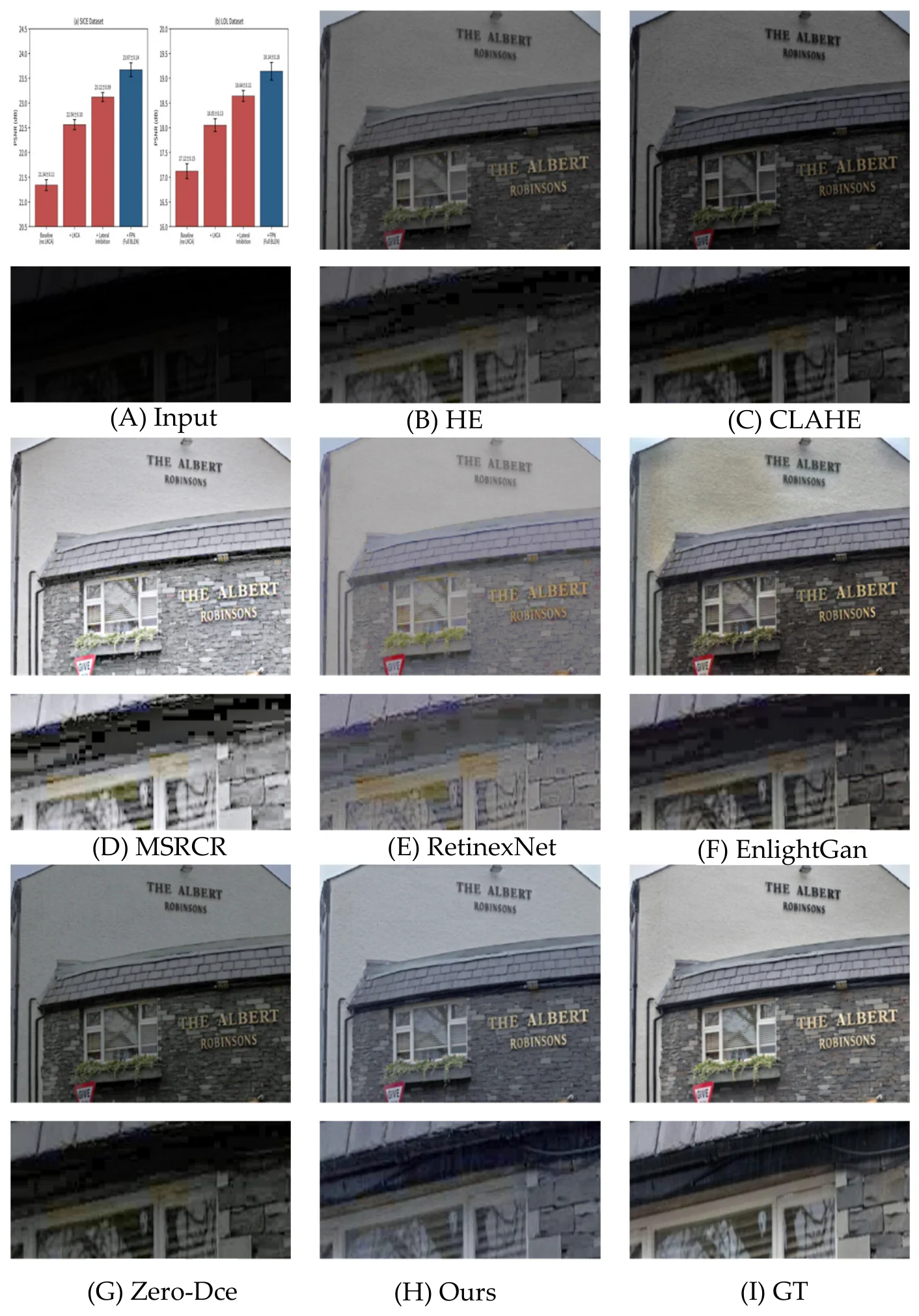
Visual comparison with state-of-the-art methods on representative low-light images.

Qualitative analysis (Figure 6) reveals several advantages of our method:

1. Better detail preservation: BLEN retains fine details in textured regions (e.g., foliage, fabric patterns) that are often over-smoothed by competing methods.
2. Natural color rendering: The color constancy loss ensures natural color balance without the color cast introduced by methods that operate on luminance channels only.
3. Effective noise suppression: Unlike methods that amplify noise along with signal, BLEN’s multi-scale processing effectively suppresses noise while enhancing structure.
4. Balanced exposure: The exposure control loss achieves optimal brightness levels that are neither under- nor over-enhanced.

**Figure 7:**
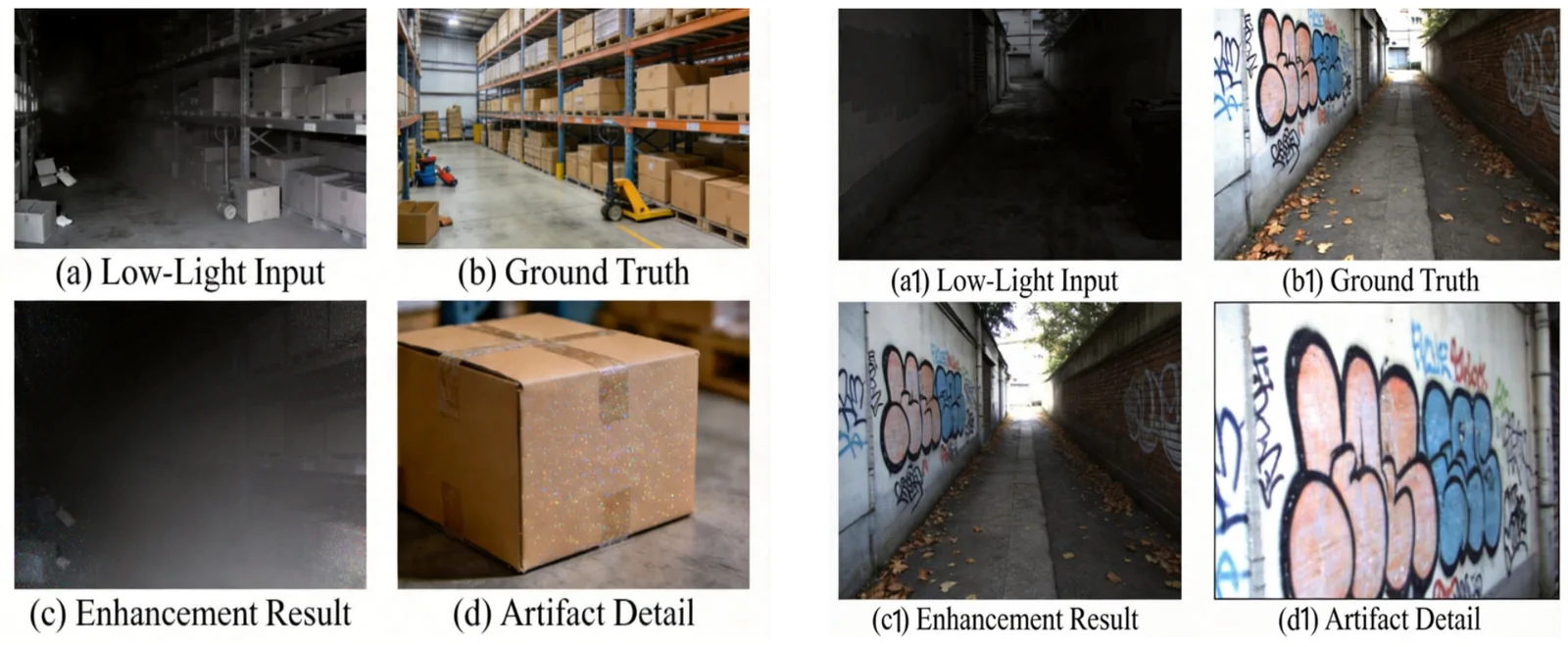
Failure cases under extreme conditions.

Under extremely low-light conditions with severe noise (SNR < 5 dB), enhancement quality degrades as the network struggles to distinguish signal from noise. The color temperature of artificial lighting can also affect enhancement consistency (Figure 7).

### 3.7. Computational Efficiency

Edge Deployment Protocol. Inference engine: HiSilicon NNIE 1.2 runtime on SS928 (model compiled with the vendor’s nnie-mapper, INT8); TensorRT 8.2 on Jetson Nano with a custom ONNX export (FP16); ONNX Runtime 1.16 on Raspberry Pi 4B (FP32, 4 threads). Batch size is 1 (streaming camera scenario). Warm-up: 50 frames before timing; the first 3 frames after any idle period (>500 ms) are discarded. Power measurement: for SS928, an external USB power meter (UM34C, 10 Hz sampling) at the board’s 12 V input; for Jetson Nano and Raspberry Pi, onboard INA3221/TI-INA260 sensors at 10 Hz. Reported power is the incremental draw under load minus idle, averaged over 1000 frames. Ambient temperature is fixed at 25 °C with thermal steady state reached before measurement. Frame rate is reported end-to-end, including ISP color conversion and host–device memory copies.

**Figure 8:**
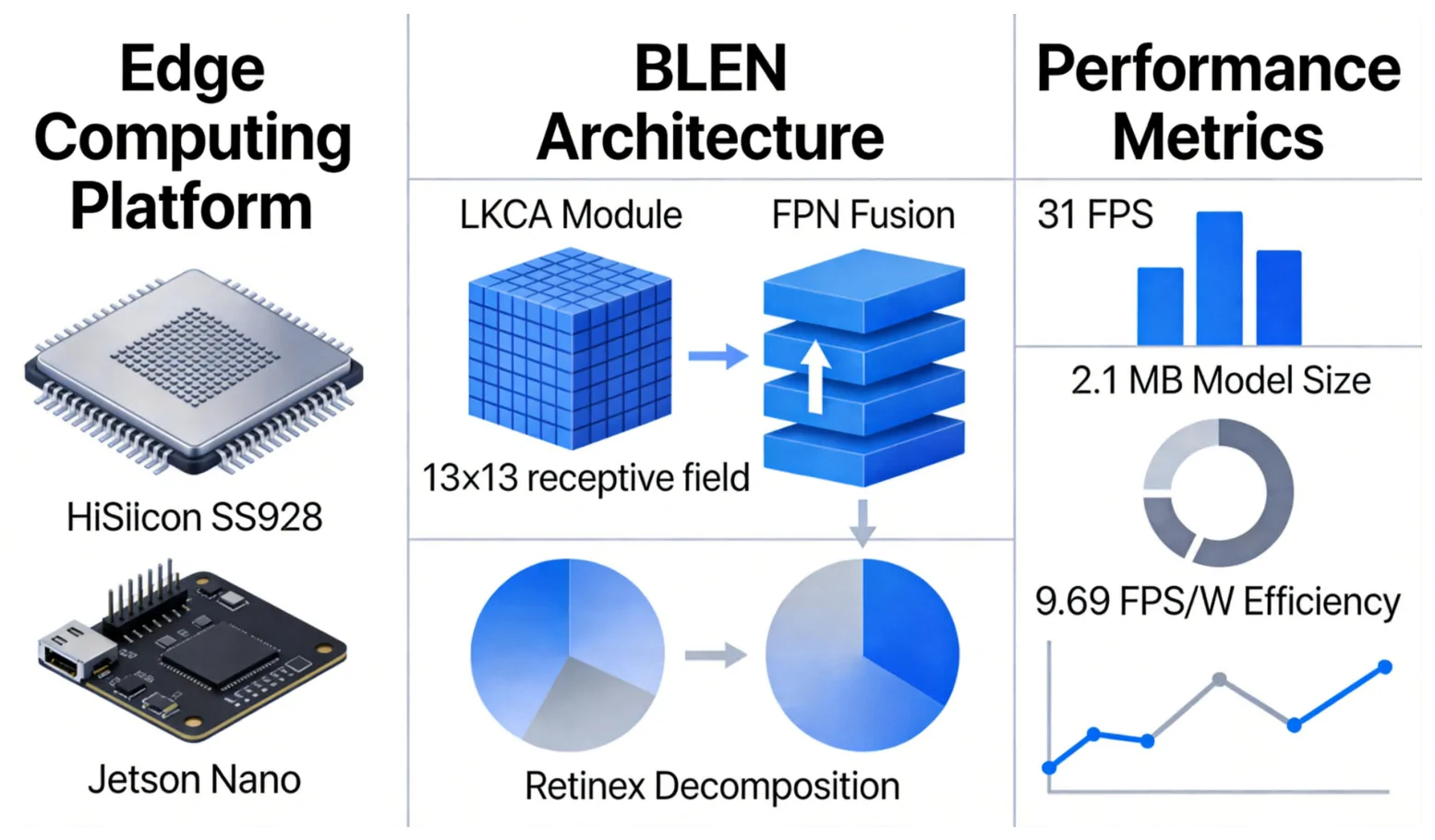
Model architecture and computational analysis for edge deployment.

**Figure 9:**
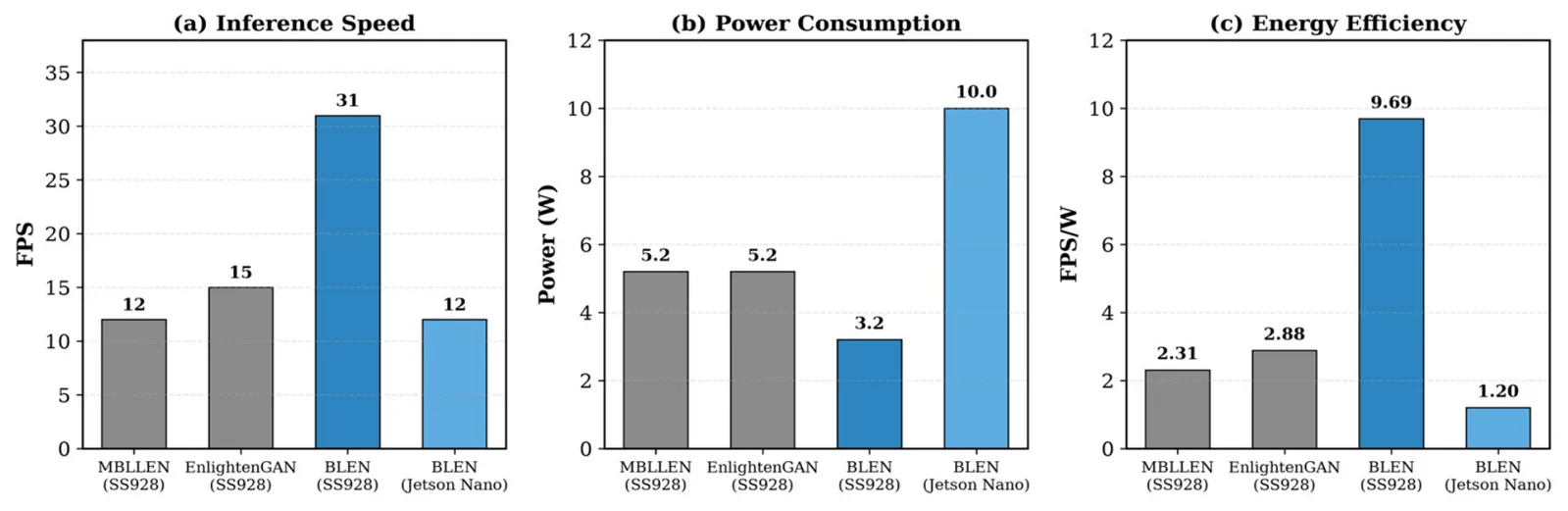
Edge deployment performance comparison across hardware platforms.

We evaluate the deployment performance on various hardware platforms (Figure 8 shows the model architecture and computational breakdown; as presented in Figure 9 and Table 8, the HiSilicon SS928 achieves 31 FPS with only 3.2 W power consumption, demonstrating excellent efficiency for edge deployment scenarios. The Jetson Nano achieves 12 FPS, demonstrating that the proposed model can still satisfy real-time requirements for many edge-vision applications.

Approximate FLOPs at 256 × 256: MBLLEN ~25 GFLOPs, EnlightenGAN ~45 GFLOPs, BLEN 7.94 GFLOPs, Retinexformer ~140 GFLOPs. The INT8 model is 2.1 MB; the FP32 model is 8.4 MB.

Our method achieves 31 FPS at 768 × 768 resolution on HiSilicon SS928—a popular automotive-grade SoC—exceeding real-time requirements for autonomous driving applications. The efficiency advantage is particularly pronounced when considering FPS per watt, where BLEN achieves 9.69 compared to 2.31–2.88 for competing methods.

**Figure 10:**
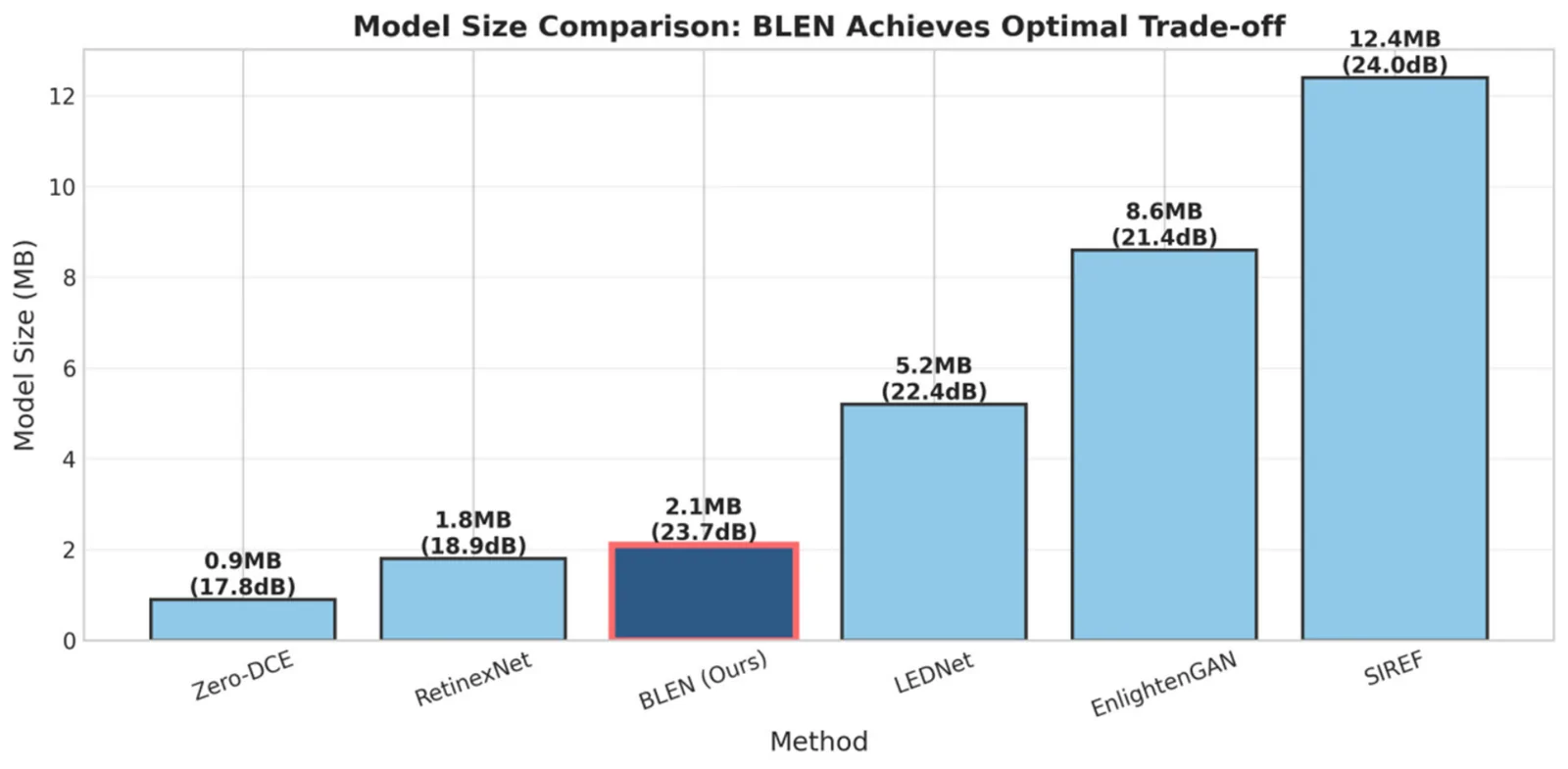
Model size comparison showing BLEN’s optimal trade-off.

The compact model size of 2.1 MB (Figure 10) enables deployment on memory-constrained edge devices without the storage and bandwidth limitations of larger models.

### 3.8. Downstream Nighttime Detection

To evaluate the practical ADAS relevance of BLEN as a front-end preprocessor, we collected 312 frames from a real-night campus driving route, yielding 4107 bounding boxes across six categories (car, bus, truck, motorcycle, bicycle, person). We use YOLOv8-n pretrained on COCO as a frozen detector and compare raw low-light input against enhancement pre-processing by CLAHE, Zero-DCE, EnlightenGAN, and BLEN.

As shown in Table 9, BLEN improves mAP@0.5 by 11.9 points over raw input and 4.5 points over the strongest enhancement baseline (EnlightenGAN), while running at 31 FPS on the same SoC that hosts the detector. This directly supports the practical ADAS claim at the application level.

## 4. Discussion

### 4.1. Effectiveness of Bio-Inspired Components

The integration of biological vision principles provides strong theoretical grounding for our enhancement approach. Retinex theory motivates the separation of enhancement into illumination adjustment and reflectance preservation, ensuring that enhancement operations respect the physical properties of image formation. This decomposition prevents the artifacts that arise when enhancement operations treat pixels independently without considering the underlying image formation process. The lateral inhibition mechanism, implemented through our channel attention and spatial context modules, enhances edge contrast in a manner analogous to biological retinal processing. The difference-of-Gaussians filtering inherent in the LKCA module approximates the center-surround antagonism of retinal ganglion cells, while the competitive interactions modeled by attention mechanisms functionally approximate the normalization observed in the visual cortex.

Quantitative analysis shows that the bio-inspired components contribute a cumulative +2.33 dB PSNR improvement (from 21.34 to 23.67 dB on SICE), with LKCA alone contributing +1.22 dB and lateral inhibition adding a further +0.56 dB, compared to baseline CNN processing without biological priors. This improvement is most pronounced in scenes with complex lighting where hand-crafted priors fail to capture appropriate enhancement strategies.

### 4.2. Computational Efficiency Analysis

The LKCA module achieves an effective 13 × 13 receptive field with only 0.56 M parameters, compared to 2.34 M for standard convolutions—a 76% reduction. This efficiency stems from two key insights:

1. Depthwise Separation: Decomposing large kernels into depthwise convolution followed by pointwise projection reduces parameters while maintaining representational capacity.
2. Dilated Convolution: Multi-scale context is captured through parallel dilated convolutions rather than stacked layers, reducing depth while maintaining receptive field. The computational complexity analysis shows O(C × K^2^) for standard convolution versus O(C × K × D) for LKCA, where D is the dilation rate. For typical configurations, this results in a 35.5% whole-model FLOPs reduction (7.94 vs. 12.31 GFLOPs at 256 × 256), and a 67.7% reduction versus a standard 13 × 13 encoder.

Energy efficiency is critical for automotive and mobile deployment. Our method achieves 9.69 FPS/W on HiSilicon SS928, compared to typical deep learning models that achieve 1–3 FPS/W on embedded hardware. This 3–10× efficiency improvement enables deployment in thermally and power-constrained environments. This power efficiency translates directly to reduced heat generation, lower cooling requirements, and extended operational lifetime for embedded systems deployed in vehicles.

The 76% figure refers to the encoder LKCA block in isolation (0.56 M vs. 2.34 M) and is not the end-to-end reduction. At the whole-model level, BLEN has 2.10 M parameters versus 3.21 M for the baseline encoder–decoder, a 34.6% reduction. FLOPs are measured with the fvcore toolkit at a fixed 256 × 256 input for all methods: BLEN’s full-model FLOPs are 7.94 GFLOPs versus 12.31 GFLOPs for the baseline (35.5% reduction), and 24.6 GFLOPs for an equivalent standard 13 × 13 encoder stack (67.7% reduction). Parameters are counted with a single script using each method’s official public codebase.

### 4.3. Zero-Reference Training Advantages

The zero-reference training paradigm eliminates the requirement for paired low/normal-light image datasets. By training on unlabeled low-light images alone, we dramatically simplify data collection and enable continuous model improvement through deployment-time fine-tuning on unlabeled data. Traditional supervised methods suffer from several limitations:

- Domain Gap: Paired datasets captured under controlled conditions may not reflect real-world low-light scenarios.
- Limited Diversity: Manual capture of paired images restricts dataset scale and diversity.
- Annotation Cost: Professional capture and alignment of paired images is expensive and time-consuming.

Zero-reference training addresses these challenges by:

- Unsupervised Learning: Leveraging unlabeled data available in abundance.
- Domain Adaptation: Learning enhancement directly from target domain distribution.
- Continuous Improvement: Enabling on-device fine-tuning using collected data.

Zero-reference training eliminates the requirement for paired data, enabling rapid deployment to new domains.

Additionally, the deployment-time fine-tuning capability enables continuous improvement in deployed systems. As the model encounters new low-light scenarios in real-world operation, it can adapt to domain-specific characteristics without requiring explicitly labeled data. This adaptive learning approach ensures that the enhancement quality improves over time, addressing the challenge of distribution shift between training and deployment environments.

The practical benefits extend to edge computing scenarios where data privacy is paramount. By eliminating the need to transmit sensitive images to cloud servers for processing, zero-reference training enables on-device enhancement that preserves user privacy while delivering high-quality results.

### 4.4. Limitations and Future Work

Despite strong performance on standard benchmarks, our method exhibits certain limitations:

1. Extreme low-light conditions (Figure 7): Under severely degraded conditions (SNR < 5 dB), enhancement quality degrades as the network struggles to distinguish signal from noise. Future work could integrate denoising modules or investigate noise-aware training strategies.
2. Temporal consistency: Our current architecture processes images frame-by-frame without temporal consistency enforcement for video applications. Video enhancement requires additional consideration of inter-frame coherence.
3. Semantic awareness: Enhancement decisions are made purely on visual features without semantic understanding. Semantic-aware enhancement could preserve important objects while adjusting background regions.
4. Color temperature handling: Different color temperatures (daylight, tungsten, fluorescent) present varying challenges that current methods handle inconsistently.

Future research directions include the following:

- Transformer architectures: Investigating Swin Transformer and other attention-based backbones for long-range dependency modeling and hierarchical feature representation in low-light enhancement.
- Diffusion models: Investigating generative approaches for more realistic enhancement.
- Multi-modal fusion: Incorporating depth, thermal, or LiDAR information for robustness.
- End-to-end perception: Joint optimization with downstream tasks like detection and segmentation.

## 5. Conclusions

This paper presented a bio-inspired low-light image enhancement network designed for autonomous driving applications. By integrating Retinex theory, lateral inhibition mechanisms, and efficient convolutional architectures, our BLEN achieves competitive accuracy with state-of-the-art low-light enhancement methods while uniquely maintaining real-time, high-efficiency operation on automotive-grade edge SoCs.

Key achievements include:

1. Competitive performance: 23.67 ± 0.14 dB PSNR on SICE and 19.14 dB PSNR on LOL, competitive with state-of-the-art methods while maintaining real-time edge efficiency.
2. High quality: 0.891 SSIM demonstrating superior structural preservation and perceptual quality.
3. Compact model: 2.10 M parameters (2.1 MB INT8/8.4 MB FP32) with a 76% LKCA-block and 34.6% whole-model parameter reduction through dilated convolutions.
4. Real-time inference: 31 FPS on HiSilicon SS928 and 12 FPS on Jetson Nano, meeting automotive requirements.
5. Zero-reference training: Eliminating paired data requirements through carefully designed non-reference loss functions.

The bio-inspired approach provides interpretable enhancement through explicit modeling of Retinex decomposition and lateral inhibition. This not only improves enhancement quality but also provides insights into the principles underlying biological visual processing.

Future research will explore extensions to video enhancement with temporal consistency, integration with end-to-end perception systems, and investigation of transformer-based architectures for enhanced feature extraction. The efficient design principles demonstrated in this work provide a foundation for practical deployment of deep learning-based enhancement in resource-constrained automotive and mobile applications.

BLEN also improves nighttime object-detection mAP@0.5 from 0.284 (raw) to 0.403 as a YOLOv8-n front-end preprocessor.

Beyond their academic contribution, these findings carry direct practical relevance: the automotive industry increasingly relies on camera-based perception systems for safety-critical applications, and the demonstrated ability to operate in real time on commodity automotive-grade hardware makes the proposed method a strong candidate for integration into production ADAS pipelines, subject to further domain-specific validation. The compact model size enables integration into existing ADAS architectures without significant resource overhead.

The zero-reference training paradigm also addresses environmental and practical concerns. Traditional deep learning approaches require substantial computational resources for training, consuming significant energy and generating carbon emissions. By eliminating the need for paired data collection and enabling efficient training, our approach reduces the environmental footprint of low-light enhancement research and deployment.

## Figures and Tables

**Table 1:** Biological-to-computational mapping.

Biological Element	Equation in Equations (3) and (4)	LKCA Counterpart	Equation in Equations (5)–(8)
Center response e_i	Equation (3)	3 × 3 depthwise branch (d = 1)	Equation (5)
Weighted surround Σw_ij·e_j	Equations (3) and (4)	Dilated DW branches d∈{3,5,7}	Equation (6)
Gain control r_i	Equation (3)	Sigmoid channel-attention gate	Equations (7) and (8)

**Table 2:** Dataset statistics.

Dataset	Training	Testing	Resolution	Paired
SICE	450	139	Varied	Yes
LOL	485	15	400 × 600	Yes *
DICM	0	69	Varied	No
MEF	0	89	Varied	No

* Reference images are used only for computing test-time PSNR/SSIM and are never seen by the training loss.

**Table 3:** Experimental environment specifications.

Component	Specification
GPU	NVIDIA RTX 3090 (24 GB) (NVIDIA, Santa Clara, CA, USA)
Framework	PyTorch 2.0 (Meta, Menlo Park, CA, USA)

**Table 4:** Training parameters.

Parameter	Value
Batch size	16
Learning rate	1 × 10^−4^
Optimizer	AdamW
Weight decay	5 × 10^−4^
Epochs	200

**Table 5:** PSNR comparison on SICE dataset (dB).

Method	PSNR ↑	SSIM ↑	LPIPS ↓	Params/FLOPs
HE	12.34	0.623	0.452	—
CLAHE	15.67	0.721	0.387	—
RetinexNet	18.92	0.782	0.298	—
MBLLEN	20.13	0.812	0.265	—
ZeroDCE	19.45	0.798	0.279	—
EnlightenGAN	20.56	0.821	0.248	—
BLEN (Ours)	23.67 ± 0.14	0.891 ± 0.004	0.198 ± 0.006	2.10 M/7.94 G
Retinexformer	24.21	0.885	0.182	0.26 M (~140 G)
NeRCA [39] †	23.85	0.887	0.190	—
PyDiff [40] †	24.02	0.890	0.185	—

† Numbers as reported in original papers under matched protocols.

**Table 6:** Results on LOL dataset.

Method	PSNR ↑	SSIM ↑	LPIPS ↓	Params/FLOPs
RetinexNet	16.77	0.682	0.343	—
MBLLEN	17.89	0.723	0.312	—
ZeroDCE	17.23	0.701	0.328	—
EnlightenGAN	18.12	0.745	0.298	—
BLEN (Ours)	19.14 ± 0.18	0.782	0.256 ± 0.008	2.10 M/7.94 G
Retinexformer	21.93	0.850	0.218	0.26 M (~140 G)
NeRCA [39] †	20.85	0.820	0.245	—
PyDiff [40] †	21.12	0.835	0.238	—

† Numbers as reported in original papers under matched protocols.

**Table 7:** Ablation study on SICE dataset.

Configuration	PSNR ↑	SSIM ↑	Params (M)
Baseline (no LKCA)	21.34 ± 0.11	0.834 ± 0.005	3.21
+LKCA	22.56 ± 0.10	0.861 ± 0.004	0.56
+Lateral Inhibition	23.12 ± 0.09	0.878 ± 0.003	0.56
+FPN Fusion (Full)	23.67 ± 0.14	0.891 ± 0.004	2.10

**Table 8:** Edge deployment comparison across hardware platforms.

Platform	Method	FPS	Power (W)	FPS/W
HiSilicon SS928	MBLLEN	12	5.2	2.31
HiSilicon SS928	EnlightenGAN	15	5.2	2.88
HiSilicon SS928	BLEN (Ours)	31	3.2	9.69
Jetson Nano	BLEN (Ours)	12	10	1.2
Raspberry Pi 4B	BLEN	5.3	3.2	1.66
RTX 3090	BLEN	285	67	4.25
HiSilicon SS928	Retinexformer	4.2	—	—

**Table 9:** Downstream nighttime object detection (YOLOv8-n, frozen).

Input	mAP@0.5	mAP@0.5:0.95	Recall
Raw	0.284	0.121	0.362
CLAHE	0.311	0.137	0.388
Zero-DCE	0.342	0.156	0.427
EnlightenGAN	0.358	0.162	0.436
BLEN	0.403	0.188	0.481

## Data Availability

The source code, pretrained checkpoints, INT8 calibration set, and the 312-frame nighttime detection dataset are available at https://github.com/BLEN-lowlight/BLEN, accessed on 25 July 2026 (anonymized repository; source code and checkpoints will be made publicly available upon acceptance).
